# Survey to inform personalised prescribing in a British South Asian community: pharmacogenomics and traditional medicine use

**DOI:** 10.1186/s12916-026-04914-9

**Published:** 2026-05-15

**Authors:** Emma F Magavern, Gabriel Marengo, Stavroula Kanoni, Mahendra G Patel, Marie Spreckley, Mehru Raza, Taleah Khan, Genes & Health Research Team, David Collier, Mark J Caulfield

**Affiliations:** 1 William Harvey Research Institute, Queen Mary University of London, London, EC1M 6BQ UK; 2 Centre for Research Equity, Nuffield Department of Primary Care Health Sciences, University of Oxford, Oxford, UK; 3 MRC Epidemiology Unit, University of Cambridge, Cambridge, CB2 0SL UK; 4 Our Future Health, Manchester, M3 5GS UK; 5 Icahn School of Medicine, Mount Sinai, NY 11373 USA; 6 Barts Clinical Trials Unit, Wolfson Institute of Population Health, Queen Mary University of London, London, EC1M 6BQ UK

**Keywords:** Pharmacogenomics, Therapeutics, Cytochrome P450, Traditional medicines

## Abstract

**Background:**

Pharmacogenomics (PGx) uses genetic information to personalize medication, reducing adverse reactions and improving efficacy. Despite its promise, low public awareness and disparities in PGx acceptability among under-represented groups may exacerbate health inequalities. The objective of this study was to elucidate a British South Asian community’s attitudes toward personalised prescribing.

**Methods:**

Adults of Bangladeshi or Pakistani ancestry from the Genes & Health (G&H) study completed a survey. Community feedback guided theme prioritization. Multivariable logistic regression analyses (controlling for age and gender) explored relationships among survey variables, and case–control Genome Wide Association Studies (GWAS) and candidate variant enrichment analysis examined the genetic architecture underlying herbal remedy use.

**Results:**

Out of 553 respondents (57% female, mostly aged 25–54), 72% reported medication inefficacy, and 54% experienced side effects. Herbal remedies were widely used (66%), notably Black seed (39%), Turmeric (37%), and Ginger (36%). Participants who reported not using traditional or herbal medicines had higher medication adherence MARS-5 scores (Odds Ratio (OR) 1.10, 95% Confidence Interval (CI) 1.05–1.16, *p* < 0.0002). All three commonly used herbal remedies inhibit the pharmacogenomically variable CYP2C9 enzyme responsible for metabolising commonly used medications. 58% of respondents were willing to provide DNA samples for PGx testing, yet 70% agreed that they would be more likely to take medication as instructed if PGx results suggested the medicine would suit them. Concerns about PGx testing were common (27%), especially among non-English speakers. Most (69%) were concerned about misuse of PGx data, particularly by pharmaceutical companies (82%). Importantly, 87% demanded stronger PGx data protections compared to other health data.

**Conclusions:**

Compared to a national UK population, the surveyed subpopulation reported higher rates of adverse drug reactions (ADRs) and perceived medication inefficacy, yet fewer respondents indicated willingness to undergo PGx testing. This highlights the need for tailored implementation strategies and underscores the importance of engaging underrepresented populations in policy development. The inverse relationship between medication adherence and herbal remedy use indicates an association between cultural health practices and medication behaviours that merits further investigation. Increased awareness of the common use of these CYP2C9 inhibitors and further research into the genetic architecture underlying herbal remedy use are warranted.

**Supplementary Information:**

The online version contains supplementary material available at 10.1186/s12916-026-04914-9.

## Background

Medication is recommended when benefits are considered to outweigh potential risks [1]. Though evidence of efficacy is required on a population level, it is understood that there is variability in medication response and not everyone will benefit from a particular medication [2]. Likewise, some patients have a higher risk of experiencing adverse drug reactions (ADRs) than others [3]. Though many factors are known to impact on this variability in medication response, including co-morbidities, age, sex, weight, and drug-drug interactions, genetic variation also plays a role [3]. This is known as pharmacogenomics (PGx). Though many variants that impact medication response are known to be more common in some biogeographic groups than others, clinical trials often extrapolate medication response data from a relatively homogenous group of participants of European ancestry, and there are concerns that without adequate engagement with underrepresented communities PGx could exacerbate existing health inequity [4]. Individuals of south Asian ancestry are known to be underrepresented in genetic research [5]. Within the United Kingdom (UK) context this is a rapidly growing and important demographic to engage with to ensure that PGx clinical implementation is fit for purpose [6]. Prior qualitative work done with the Genes & Health study cohort of British participants of Bangladeshi and Pakistani ancestry highlighted many interesting themes that merited further exploration. It shone a spotlight on the importance of trust in successful implementation of PGx and the crucial role education, communication, and outreach play in facilitating trust [7]. Yet there remains a need to quantify this feedback to support equitable and inclusive PGx clinical implementation in the UK. Furthermore, prior research has shown that traditional and herbal medicines are commonly used by the south Asian diaspora in the UK, yet most patients using these medicines alongside prescription medications do not divulge this information to their healthcare practitioner [8, 9]. It is not clear if or how genetic variability to medication response may overlap with the use of traditional and herbal medicines, many of which are known to interact with prescription medications.

Despite growing awareness of PGx variability and the common use of herbal medicines among South Asian populations in the UK, there is a lack of research exploring how these two domains overlap in real-world patient behaviours [8]. Specifically, little is known about whether traditional remedy use might interact with genetically predicted drug metabolism — particularly for enzymes such as CYP2C9 which are both pharmacogenetically variable and inhibited by common traditional compounds. Furthermore, community-specific attitudes and beliefs around PGx remain under-characterised in a quantifiable way.

This study addresses this gap by quantifying PGx attitudes in a cohort of British Bangladeshi and Pakistani individuals already engaged in genomic research, while also exploring traditional medicine use and its potential intersection with PGx. We are not aware of any studies using either a candidate gene approach to assess herbal remedy usage or conducting Genome Wide Association Studies (GWAS) with remedy use as a trait. Such an approach is relevant considering the under exploration of remedy use and may both elucidate the genetic architecture of remedy use and highlight shared metabolism pathways with known medications. To our knowledge, this is the first study to assess these dimensions together, with implications for both precision medicine and culturally responsive PGx implementation.

## Aim

To quantify attitudes toward personalised medicine, including traditional and herbal medicine use, in a British South Asian community, and thereby support inclusive and fit for purpose clinical implementation.

## Methods

### Genes & health

Genes & Health (G&H) is a community genetics study including participants of self-identified Bangladeshi and Pakistani ancestry aged 18 and older [10]. Saliva collected during recruitment has undergone genotyping array analysis and whole exome sequencing. Participant recruitment and genetic data was linked with hospital episode statistics and primary care records. Participants consented to be re-contacted and many supplied contact e-mails.

### Survey design

**Fig. 1:**
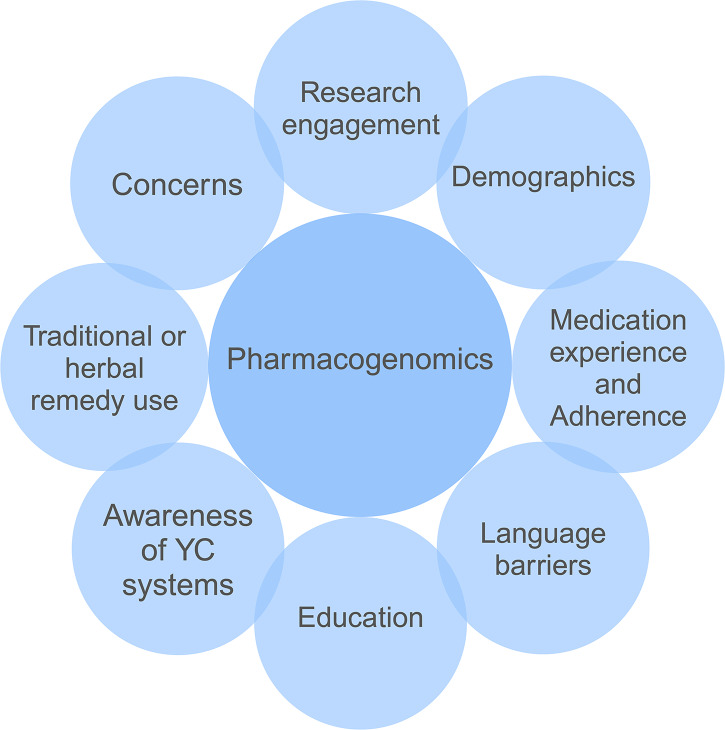
Themes explored in the survey. YC = Yellow Card

A survey was designed to quantify views within the G&H community around the themes which emerged from four intensive community-based focus group session and extensive discussion with the Community Advisory Board and multilingual community engagement team [7]. Themes of particular interest to the community and team were included such as: the intersection of PGx with medication use and adherence, experience with ADRs, data sharing and trust, traditional and herbal medicines use, and education. This study was designed to characterize awareness of PGx, facilitators and barriers to PGx implementation, and attitudes toward clinical PGx data sharing for research. The themes generated by the focus groups and explored in the survey are shown in Fig. 1. The survey was designed in partnership with the G&H engagement team members and Community Advisory Board as well as external advisors and citizen scientists. A validated self-reported adherence tool, the MARS-5, was used to assess medication adherence [11]. A full version of the survey can be found in Additional File 1. The survey was in English.

### Survey platform and dissemination

The survey was programmed on the Qualtrics platform and required 15 min to complete. Participants received a 10 GBP voucher upon survey completion as a ‘thank you’ for their participation. Participants from the G&H study with a listed e-mail address were selected at random with a weighted demographic sample to ensure that representative numbers of individuals across gender, age range and ethnicity were invited. Invitations were subsequently weighted to compensate for lower response rates from men than women. Invitations were e-mailed out in a staggered fashion, starting from May 8th 2024 with the last invitations sent on June 13th 2024. Participants were sent reminders to complete the survey and the survey was closed when 500 complete responses were received (further responses were included subsequently that had been started but not completed when the survey closed).

### Statistical analysis

All analysis of survey response data was done in RStudio [12]. Multivariable logistic regression analyses were used to analyse relationships between selected survey reported variables, controlled for age range and gender. Free text responses were reviewed in full and analysed inductively using content analysis. Key themes were identified through iterative comparison across responses.

### Genetic analysis of traditional or herbal remedy use

To investigate the genetic architecture of traditional or herbal remedy use, case control Genome Wide Association Studies (GWAS) were conducted with Regenie for the following cohorts: (1) comparing those participants who reported any remedy use as compared with those who did not, (2) participants who reported use of Black seed as compared with those did not, (3) participants who reported use of Ginger as compared with those who did not, (4) participants who reported use of Turmeric as compared with those who did not, (5) participants who reported use of Turmeric, Black seed or Ginger as compared with those who used none of these three remedies. Age, sex and the first 20 principal components were included as co-variates. Variants used for analysis were restricted to minor allele count (MAC) > = 10, minor allele frequency (MAF) > = 0.05 and absolute beta value of < 4. We chose to analyse use of these three remedies (Turmeric (*n* = 204 respondents), Ginger (*n* = 199 respondents), Black seed (*n* = 220 respondents)) based on respondent numbers with approximately 200 or more participants reporting use of each of these three herbal remedies. Top hit variants were identified by pruning p-value ≤ 5e-05 at a +/-500 kb (1 MB in total) window and top variants per locus were annotated with the nearest gene.

An enrichment study was conducted to compare genetically predicted CYP2C9 enzyme poor or intermediate metabolisers with normal metaboliser participants in subgroups using traditional or herbal remedies which impact on these metabolism pathways, using Fisher’s exact tests. Decreased function variants in *CYP2C9*, the gene encoding the CYP2C9 enzyme, are known to be common in South Asian populations, and many of the commonly used supplements are known to inhibit CYP2C9 function. The CYP2C9*2 (NG_008385.2:g.9133 C > T) and CYP2C9*3 (NG_008385.2:g.48139 A > C) alleles are well validated decreased function variants known to be common in south Asian populations and we thus focused on these variants, using the genetic diplotype to infer CYP2C9 metaboliser phenotype based on consortia guidance [13–15].

## Results

553 participants consented to the study and completed some of the survey, with 508 respondents completing the whole survey in response to 4860 invitations to valid e-mails (11% study participation rate). A full summary of responses to the survey questions can be found in Additional File 2.

### Demographics

Demographic information is shown in Table 1. 57% of respondents were female and most respondents were between 25 and 54 years old. 67% had completed a university degree (most commonly in the UK), and the most common primary spoken language was English (72%). 70% of respondents were in paid employment.

**Table 1:** Survey respondent demographic characteristics (*n* = 508)

Gender	Male Female	43% 57%	290 218
Age (years)	18–24 25–34 35–44 45–54 55–64 65–74 Over 74	17% 27% 28% 20% 5% 2% 1%	84 139 142 101 27 11 4
Education level	No schooling completed Primary school Secondary school Technical college qualification University degree	1% 12% 20% 67%	4 3 59 102 340
Country of Education	UK Pakistan Bangladesh India EU country Other	83% 6% 8% 0% 1% 1%	423 33 39 2 7 4
Primary Language	Balochi Bengali English Kashmiri Punjabi Pashto Sylheti Urdu Other	0% 12% 72% 0% 4% 1% 1% 9% 1%	1 60 368 1 18 4 6 46 4
Employment	Yes No	70% 30%	368 150

Survey response numbers are shown in the right column with percentage on the left column

### Awareness of factors impacting variability in medication response

Most respondents were aware that age (78%), sex (57%), weight (68%), co-morbidities (90%), drug-drug interactions (77%), or exposures like smoking and alcohol (71%) can impact on medication response. Interestingly, though 67% of respondents identified DNA or genetics as impacting on medication response, fewer (55%) thought ethnicity may play a role and fewer still thought that the response of a family member to the same medication was relevant (31%). 63% agreed that DNA can influence response to medications.

72% of respondents reported taking a medication which they felt did not work for them, with 54% reporting that they had experienced a side effect from a medication. 47% of respondents were prescribed medication on a regular basis, and the average number of medications taken was three.

### MARS-5- medication adherence report scale

39% of respondents reported sometimes (23%), often (10%) or always (6%) taking less medication than instructed, while 45% reported they sometimes (29%), often (13%) or always (3%) stop taking medication for a while. 43% of respondents reported sometimes (31%) often (11%) or always (1%) missing out a dose. 18% of participants reported that they alter the dose sometimes (14%), often (3%), or always (1%), while 54% sometimes (39%), often (12%) or always (3%) forget to take medication. 5-item Medication Adherence Report Scale (MARS-5) scores, which measure adherence, with a maximum possible score of 25 (perfect adherence) ranged from 10 to 25 (1st quartile 16, Median 19, Mean 18.97, 3rd quartile 22, Max 25).

### Traditional or herbal remedy use

Only 34% of respondents reported not using any traditional or herbal remedies. Among the listed agents, the most commonly used were Black seed (Kalonji/Kalojeera) (39%), Turmeric home remedies (Haldi) (37%), Ginger home remedies (36%), Fennel (Saumf) (22%), Psyllium husk (Isabgol/Isapgol) (18%), and Joshanda (17%). 32% of respondent sometimes (20%), often (10%) or always (2%) used these traditional or herbal remedies, with 37% reporting only occasional use. Of respondents who said they at least occasionally used traditional or herbal remedies, 77% of respondents reported that they used these alongside prescribed medicine, while 14% reported that they use these remedies instead of prescribed medicine. Participants with higher self reported medication adherence (MARS-5 scores) were more likely to report not using traditional or herbal medicines (Odd Ratio (OR) 1.10 per 1 point increase in adherence scale, 95% Confidence Interval (CI) 1.05-1.16, *p* <0.0002 in logistic regression analysis with age and gender as covariates.

### Desirability of PGx testing

**Fig. 2:**
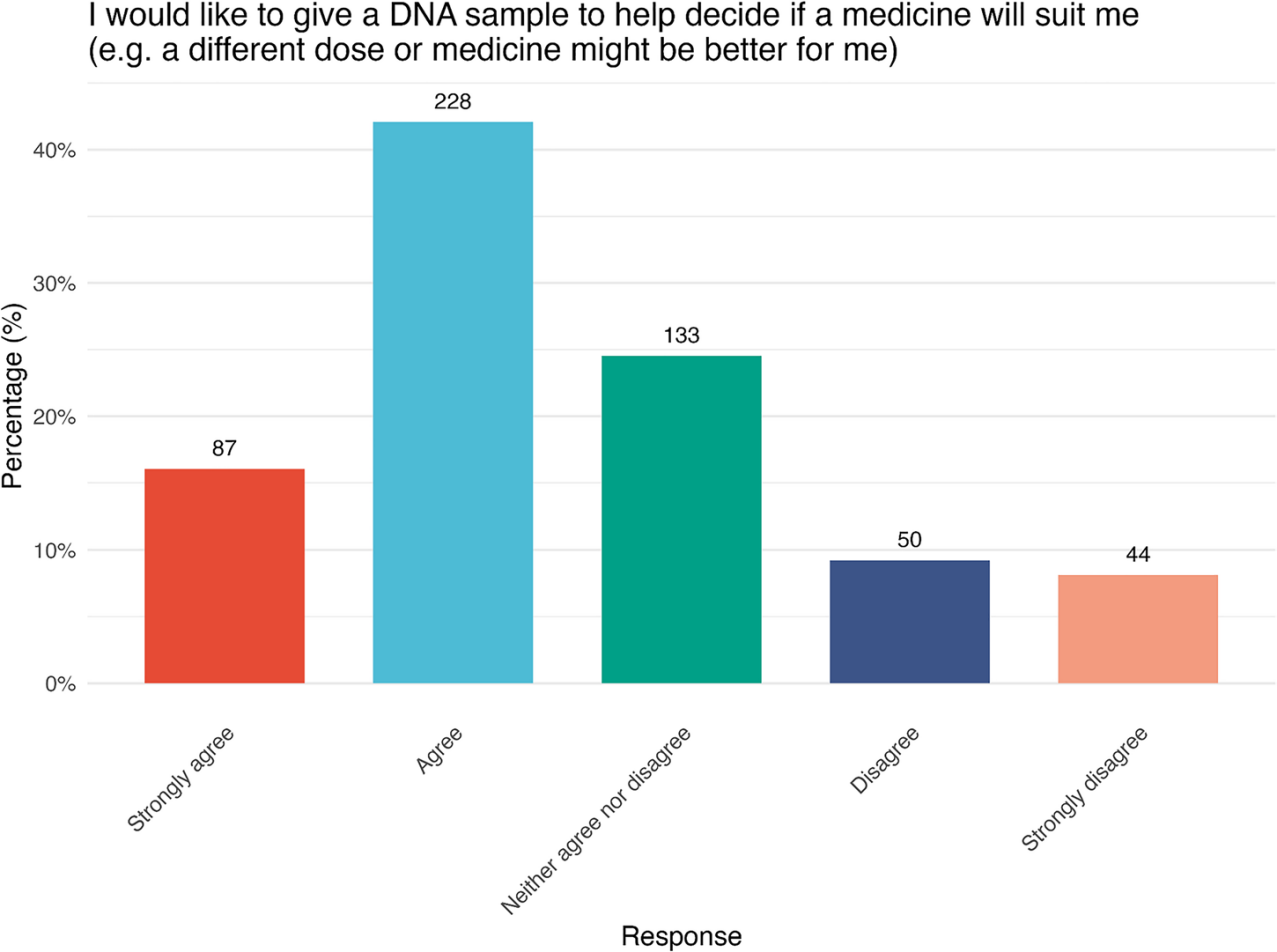
Respondent willingness to undertake clinical PGx testing

**Fig. 3:**
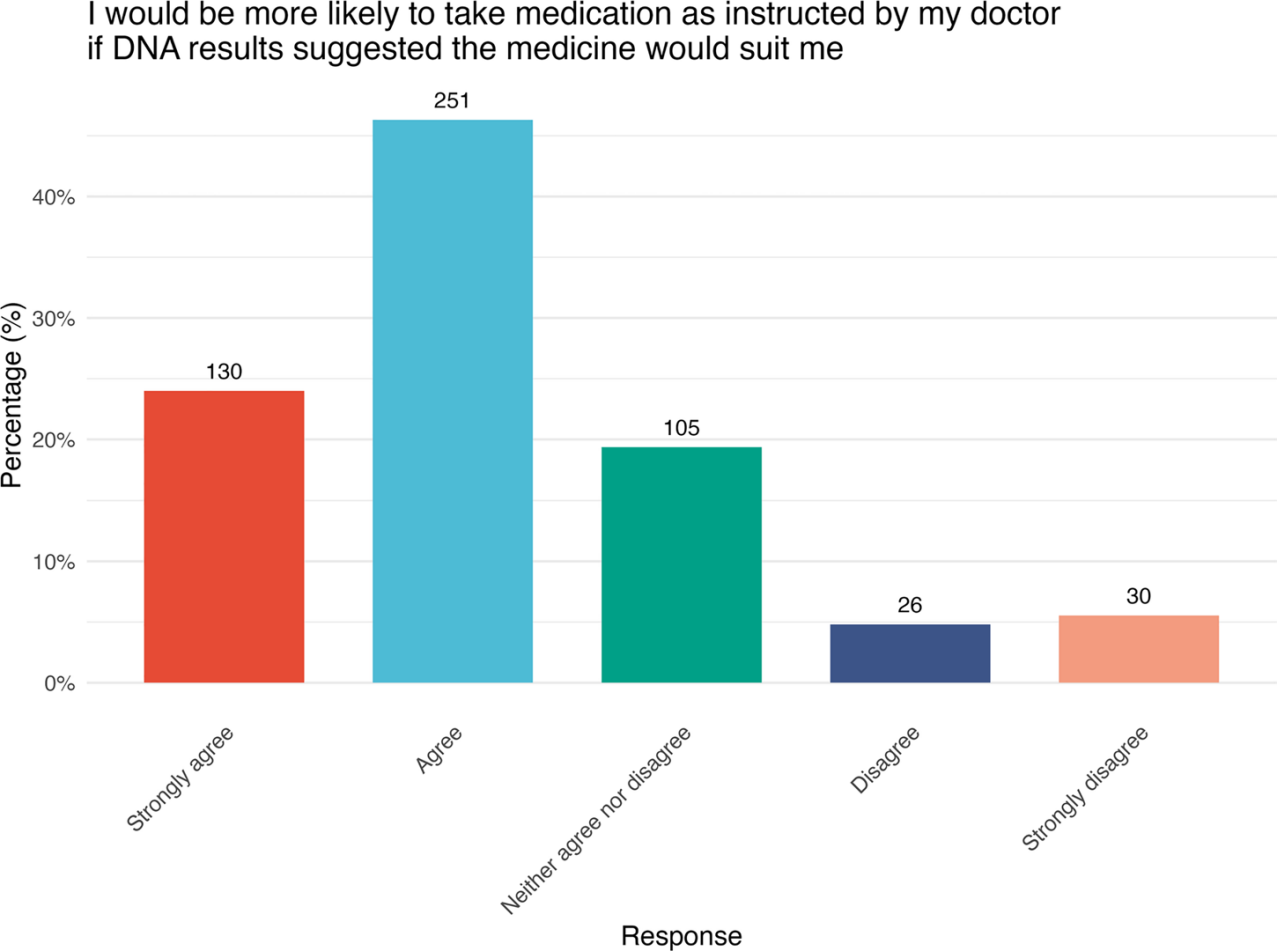
Respondent perception of impact of PGx testing on medication adherence behaviour

58% of respondents agreed (42%) or strongly agreed (16%) that they would like to give a DNA sample to help decide which medicine would best suit them, yet 70% either agreed (46%) or strongly agreed (24%) that they would be more likely to take medication as instructed if DNA results suggested the medicine would suit them (Figs. 2 and 3). There was no significant association between attitudes toward PGx impact on future adherence and MARS-5 self-reported medication adherence scores (OR 1.02, 95% CI 0.97–1.07, p 0.54).

### Concerns about clinical PGx testing

**Fig. 4:**
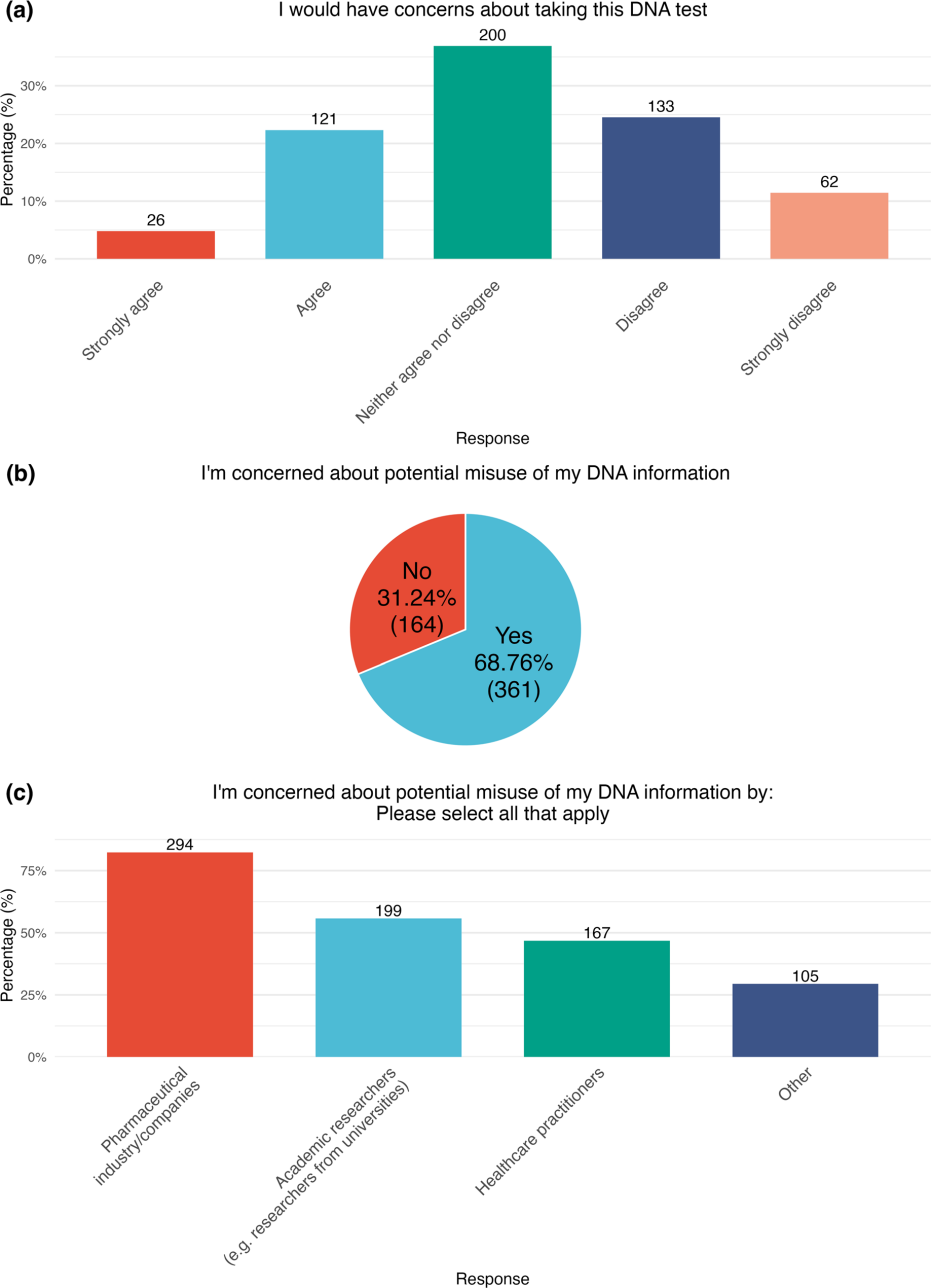
Respondent concerns around PGx testing and potential misuse of PGx data

27% of respondents either agreed (22%) or strongly agreed (5%) that they would have concerns about taking a DNA test in this PGx context (Fig. 4A). Free text comments (*n* = 305 respondents) involved concerns that appropriate medicine would not be given because of PGx test results, worries about data misuse and sharing (with police, government, or private commercial entities), incidental results, the sampling procedure, data storage, confidentiality, lack of trust, privacy, concerns about data being used to target specific populations, and the strength of evidence behind personalising prescribing with DNA tests. 53% of respondents either agreed (42%) or strongly agreed (11%) that they would encourage family or friends to have a DNA test to guide medicine use. Those participants who reported English as their primary spoken language were less likely to have concerns about taking a PGx test (OR 1.75 for disagree or strongly disagree with concerns about taking PGx test, CI 1.13–2.75, p 0.013).

### PGx data custodianship

72% of respondents either agreed (54%) or strongly agreed (18%) that they would like to hold this PGx testing information about how they might respond to medications themselves. Similarly, 74% of respondents either agreed (52%) or strongly agreed (22%) that they would like this information to be kept in hospital and GP NHS healthcare records.

### Clinical PGx data sharing for research purposes

While 74% of respondents were willing to share information from their DNA about medication response with the UK Medicines regulators and 66% with academic researchers, the number was lower (44%) for pharmaceutical industry researchers. 23% of respondents either agreed (18%) or strongly agreed (5%) that they would be more comfortable sharing this information for research if a faith leader had done so, while 35% of respondents either agreed (29%) or strongly agreed (6%) that they would be more comfortable sharing this information if family or friends had done so.

69% of respondents were concerned about potential misuse of DNA information in the context of PGx testing (Fig. 4B). For those participants with concerns, concerns were highest with pharmaceutical industry (82%), with a significant proportion concerned about misuse by academic researchers (56%) or healthcare practitioners (47%) (Fig. 4C). 87% of respondents said they would want stronger protections on DNA information about medicine response than other health information (for example medical problems, scans or which medications they’ve been prescribed). Free text responses (*n* = 316 respondents) explaining participants wish for stronger protection included concerns about privacy, data misuse, identifiability, and wish for additional data security to prevent cybercriminals from accessing information or information from being shared without their consent. 37% of respondents either agreed (31%) or strongly agreed (6%) that there are concerns unique to the Pakistani or Bangladeshi ancestry population within the UK regarding this use of DNA to personalise medicines. Free text responses (*n* = 258 respondents) explaining such concerns highlighted worries about information being misused or used to target their community, contributing to bias/discrimination, misinformation, health inequalities, lack of awareness of PGx and genetics generally, mistrust in government and healthcare from the covid-19 pandemic, and concerns about language barriers.

### Education

75% of respondents either agreed (53%) or strongly agreed (22%) that educating patients about this use of DNA testing (to tailor medication choices rather than to look for disease) is important. Respondents predominantly wanted to receive information via GP survey/hospital clinic leaflets (77%) or posters (66%), with 54% selecting social media, 47% community centres, 45% schools, 43% mosque and 40% videos as preferred dissemination avenues. Primary English speakers were more likely than other participants to disagree or strongly disagree that educating patients about this use of DNA testing (to tailor medication choices rather than to look for disease) is important (OR 1.75, CI 1.13–2.75 p 0.013) in logistic regression analysis with age category and gender as co-variates.

### Yellow card ADR reporting awareness

21% of respondents had heard of the Yellow Card reporting system while 79% had not. Of the subset of participants who were aware of the Yellow Card reporting system, 75% knew that anyone can submit a Yellow Card report to the UK medicines regulators to report a bad reaction to a medication.

### Genetic analysis of supplement use

**Fig. 5:**
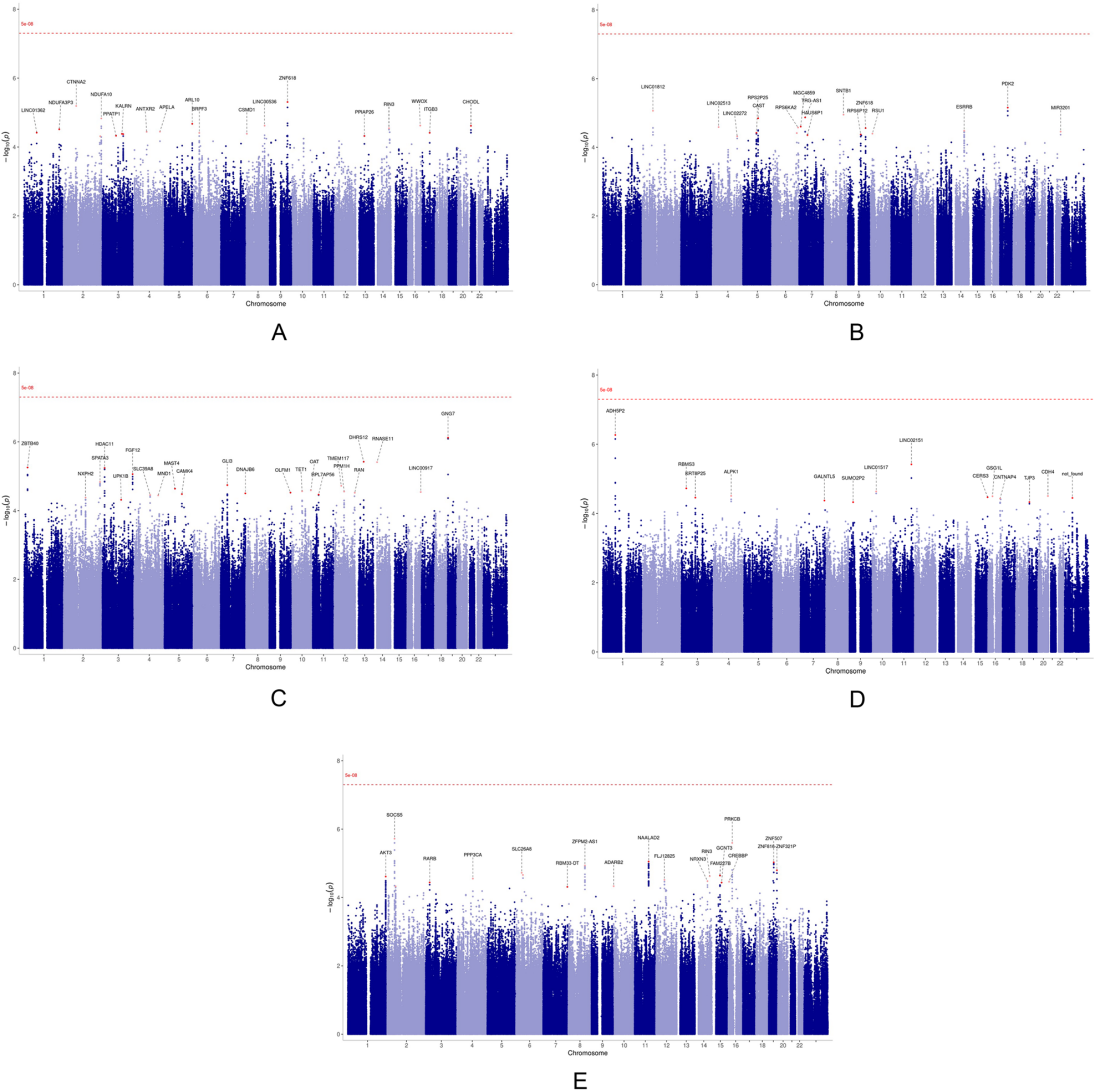
Manhattan plots of case control GWAS to examine the genetic architecture of traditional or herbal remedy use in the following groups: (**A**) participants who reported traditional or herbal remedy use as compared with those who did not (**B**) participants who reported use of Black seed as compared with those who did not (**C**) participants who reported use of Ginger as compared with those who did not (**D**) participants who reported use of Turmeric as compared with those who did not (**E**) participants who reported use of Turmeric, Black seed or Ginger as compared with those who did not. Genes are annotated at the threshold of p value ≤ 5e-05

The Manhattan plots for the conducted GWAS are shown in Fig. 5 below. 505 participants were included in the following GWAS (those who had responded to the survey and had genetic array data available). The top loci for use of traditional or herbal remedies (*n* = 329 respondents) as compared with no use of traditional or herbal remedies (*n* = 176 respondents) are shown in Fig. 5A. The top loci for use of Black seed (*n* = 198 respondents) as compared with no use of Black seed (*n* = 307 respondents) are shown in Fig. 5B. The top loci for use of Ginger (*n* = 176 respondents) as compared with no use of Ginger (*n* = 329 respondents) are shown in Fig. 5C. The top loci for use of Turmeric (*n* = 179 respondents) as compared with no use of Turmeric (*n* = 326 respondents) are shown in Fig. 5D. The top loci for use of either Turmeric, Black seed or Ginger (*n* = 286 respondents) as compared with no use of Turmeric, Black seed or Ginger (*n* = 219 respondents) are shown in Fig. 5E. These GWAS analyses presented above were exploratory with limited sample size.

The candidate gene analysis of *CYP2C9* *2 and *3 variants to infer CYP2C9 genetically predicted metaboliser phenotypes did not show enrichment of poor or intermediate CYP2C9 metabolisers in any subgroup using/not using traditional or herbal remedies which inhibit the CYP2C9 metabolism pathway (Additional file 3).

## Discussion

While this survey population was younger and had slightly higher percentage of female respondents (57% compared to 53%) than the recent UK national survey on PGx attitudes, it is interesting to compare PGx attitudes within this subpopulation to the representative national views [16].

A higher percentage of respondents reported experiences with both medication inefficacy (72% compared to 45%) and ADRs (54% compared to 46%) with respect to the representative national survey [16]. This younger population included a smaller percentage of people prescribed medications regularly (47% compared to 56% nationally) and the average number of medications prescribed was 3 as compared with 4 medications [16].

### Desirability of PGx testing

The 58% of respondents who agreed that they would like to give a DNA sample to help decide which medicine would best suit them was lower than the national response which suggested 89% of participants would complete a PGx test, though the questions were formulated differently [16]. This may suggest that pharmacogenomic uptake would be lower in this population demographic as compared with the UK national population as a whole. However, a discrete choice experiment estimated a range of potential PGx service uptake from 51% to > 99% based on optimisation of turnaround time, data availability and type of test [17]. While the respondents in this study were predominantly (90%) White British ethnicity, it is reasonable to assume there may be significantly higher uptake across ethnic groups if a hypothetical service were optimised to patient preference. The 70% of respondents who agreed that adherence would improve with PGx testing concurs with the 67% of respondents in the national survey who said they would be more likely to adhere to therapy with PGx testing then without [16]. From this study it doesn’t appear that perception of PGx impact on adherence was associated with self-reported adherence behaviours.

### Concerns about clinical PGx testing

As with the UK national survey, a substantial portion of respondents had concerns about PGx testing [16]. Many of these concerns were around data security and data sharing. Similar to the national representative survey, where willingness to share data for research was lowest with private companies, concerns were highest about potential for misuse by private industry [16]. Though specific concerns highlighted around data protection were similar to those highlighted by prior studies, there is an important lesson to be heeded in that more than one in every three respondents felt that certain concerns were unique to the surveyed ancestry population [18, 19]. This emphasises the importance of policy engagement with this underrepresented ancestry group and others to identify concerns and address them with legislation and policy prospectively. Prior comparison of ethnic group concerns regarding PGx has been mainly in the USA, comparing attitudes of Caucasian with African American participants. These studies have found that non-white participants have more concerns around PGx testing, specifically around privacy and discrimination [20, 21].

### Language barriers and PGx

It is notable that primary English speakers were more likely to think that educating patients about PGx was not important and less likely to have concerns around PGx testing. This points at the need to specifically tailer outreach initiatives to patients and the public with language barriers. Community leaders, or others embedded within communities are best placed to support outreach initiatives to build on trust in working relationships [22]. While prior research in genetic counselling has shown that self-reported English competency in a USA population was associated with increased awareness and uptake of genetic testing, such findings are in the context of diagnostic rather than PGx genetic testing [23]. Given that PGx testing is less commonplace in routine clinical care than diagnostic testing, initiatives assessing understanding using optimised lay language are likely to be important generally, but specifically in populations with language barriers [24].

### Yellow card ADR reporting awareness

As compared with the UK nationally representative survey, fewer respondents (21% compared to 25%) were aware of the Yellow Card reporting system [16]. Fewer of those participants who were aware of the Yellow Card reporting system were aware that anyone can submit a Yellow Card report to the UK medicines regulators to report a bad reaction to a medication (75% compared to 88%) [16]. These statistics highlight the need to target ADR reporting awareness to underrepresented demographics.

### Traditional or herbal remedy use

It is interesting that most (66%) participants had used traditional or herbal remedies, and that the participants who did not use such remedies reported higher medication adherence behaviours. Further studies should pursue this avenue of investigation.

All three of the reported most commonly used remedies, Black seed (Kalonji/Kalojeera) (39%), Turmeric home remedies (Haldi) (37%), and Ginger home remedies (36%), are known to inhibit the Cytochrome P450 family 2 subfamily C member 9 (CYP2C9) hepatic enzyme [25–27]. CYP2C9 is an enzyme responsible for metabolising many commonly used medications such as nonsteroidal anti-inflammatories like ibuprofen, phenytoin, and warfarin [28, 29]. Therefore, there could be drug-drug interactions with conventional medications for those using these traditional and herbal remedies alongside prescription medication (most of the participants surveyed in this study). Notably, the gene encoding the CYP2C9 enzyme (*CYP2C9*) is polymorphic and decreased and loss of function (LoF) variants, which lead to a poor or intermediate CYP2C9 metaboliser phenotype, are common across all populations, but particularly common in South Asian biogeographic groups [13, 30]. These data suggest that supplement use should be considered in this demographic, particularly when a CYP2C9 metabolised medication is recommended. There remains an open question as to whether slow CYP2C9 metaboliser status may have implications in this population, given that LoF variants in the encoding gene are enriched and much of the population report taking further CYP2C9 inhibiting supplements.

### Genetic analysis of supplement use

While the numbers of participants we had available to investigate the genetic architecture of these traditional and herbal remedy uses in the GWAS analysis was limited and thus none of the signals reached genome wide significance, there are a number of suggestive signals that should be further explored in better powered studies. Such further research may provide some novel mechanistic insight into the use and action of these supplements.

### Limitations

Though the number of respondents to this survey was modest, it represents incremental gains in the area from prior published studies. This G&H population is a select group of participants who have already chosen to participate in a community genetics study, therefore respondents may not be representative of the British Bangladeshi or Pakistani populations. There is also likely to have been response bias where individuals more interested and potentially more knowledgeable about this subject area may have been more likely to participate in the study. Furthermore, the title of the study and patient information sheet may have increased participants’ reported awareness of PGx at baseline.

## Conclusions

This survey of a subgroup of the UK population of Bangladeshi or Pakistani ancestry shows that efforts to engage this underrepresented group in PGx should be undertaken prospectively to integrate a successful clinical PGx service in the UK. Concerns should be addressed at the level of national policy and robust culturally sensitive and targeted efforts should be made, especially where language barriers exist. It is notable that the majority of respondents wanted stronger protections on PGx data than other non-genetic clinical medical data. There should be increased awareness of the common use of CYP2C9 inhibiting traditional and herbal remedies and further studies to understand the genetic architecture of such remedy use.

## Supplementary Information

Below is the link to the electronic supplementary material.

Supplementary Material 1: Additional File 1: Full Survey

Supplementary Material 2: Additional File 2: Survey Results

Supplementary Material 3: Additional File 3: CYP2C9 LOF enrichment analysis

## Data Availability

The survey data, responses and the CYP2C9 LOF enrichment analysis figures are available in full in the supplementary materials. All Genes & Health data can be accessed by application to the study access team [https://www.genesandhealth.org/research/scientists-using-genes-health-scientific-research] **.**.

## Declarations

### Patient and Public Involvement

We engaged with the G&H community advisory board, engagement team and participants to design this study.

## Abbreviations

PGx: Pharmacogenomics
G&H: Genes & Health
GWAS: Genome Wide Association Studies
ADRs: Adverse Drug Reactions
UK: United Kingdom
MAC: Minor Allele Count
MAF: Minor Allele Frequency
MARS-5: 5-item Medication Adherence Report Scale
OR: Odds Ratio
CI: Confidence Interval
CYP2C9: Cytochrome P450 family 2 subfamily C member 9
LoF: Loss Of Function

## References

[1] How EMA evaluates medicines for human use European Medicines Agency. How EMA evaluates medicines for human use. https://www.ema.europa.eu/en/about-us/what-we-do/authorisation-medicines/how-ema-evaluates-medicines-human-use#related-content-19198. Accessed 3 Feb 2026.

[2] Schork NJ. Personalized medicine: Time for one-person trials. Nature. 2015;520:609–11. 25925459 10.1038/520609a

[3] Pirmohamed M. Pharmacogenomics: current status and future perspectives. Nat Rev Genet. 2023;24:350–62. 36707729 10.1038/s41576-022-00572-8

[4] Magavern EF, Gurdasani D, Ng FL, Lee SS. Health equality, race and pharmacogenomics. Br J Clin Pharmacol. 2021. 10.1111/bcp.14983. 34251046 10.1111/bcp.14983 PMC8752640

[5] Sirugo G, Williams SM, Tishkoff SA. The Missing Diversity in Human Genetic Studies. Cell. 2019;177:1080. 31051100 10.1016/j.cell.2019.04.032

[6] (2022) Ethnic group, England and Wales: Census 2021: The ethnic groups of usual residents and household ethnic composition in England and Wales, Census 2021 data.

[7] Magavern EF, Durrani F, Raza M, Lerner R, Islam MR, Clinch M, Caulfield MJ. British South Asian ancestry participants views of pharmacogenomics clinical implementation and research: a thematic analysis. Pharmacogenomics J. 2023;23:185–94. 37907686 10.1038/s41397-023-00317-8 PMC10661738

[8] Bhamra SK, Slater A, Howard C, Johnson M, Heinrich M. The Use of Traditional Herbal Medicines Amongst South Asian Diasporic Communities in the UK. Phytother Res. 2017;31:1786–94. 28905437 10.1002/ptr.5911

[9] McInnes G, Lavertu A, Sangkuhl K, Klein TE, Whirl-Carrillo M, Altman RB. Pharmacogenetics at Scale: An Analysis of the UK Biobank. Clin Pharmacol Ther. 2021;109:1528–37. 33237584 10.1002/cpt.2122 PMC8144239

[10] Finer S, Martin HC, Khan A, et al. Cohort Profile: East London Genes & Health (ELGH), a community-based population genomics and health study in British Bangladeshi and British Pakistani people. Int J Epidemiol. 2020;49:20–i21. 31504546 10.1093/ije/dyz174 PMC7124496

[11] Chan AHY, Horne R, Hankins M, Chisari C. The Medication Adherence Report Scale: A measurement tool for eliciting patients’ reports of nonadherence. Br J Clin Pharmacol. 2020;86:1281–8. 31823381 10.1111/bcp.14193 PMC7319010

[12] RStudio Team. (2022) RStudio: Integrated Development Environment for R.

[13] PHARMGKB PHARMGKB. Gene-specific Information Tables for CYP2C9, CYP2C9 Frequency Table. https://www.pharmgkb.org/page/cyp2c9RefMaterials. Accessed 6 Mar 2025.

[14] PHARMGKB: CYP2C9*2. https://www.pharmgkb.org/haplotype/PA165816543. Accessed 18 Mar 2025.

[15] PHARMGKB: CYP2C9*3. https://www.pharmgkb.org/haplotype/PA165816544. Accessed 18 Mar 2025.

[16] Magavern EF, Marengo G, Sivathasan C, et al. A United Kingdom nationally representative survey of public attitudes towards pharmacogenomics. QJM: Int J Med. 2025. 10.1093/qjmed/hcaf035. 10.1093/qjmed/hcaf035 PMC12419794 39971322

[17] McDermott JH, Sharma V, Newman WG, Wilson P, Payne K, Wright S. Public preferences for pharmacogenetic testing in the NHS: Embedding a discrete choice experiment within service design to better meet user needs. Br J Clin Pharmacol. 2024;90:1699–710. 38616172 10.1111/bcp.16058

[18] Howard HC, Joly Y, Avard D, Laplante N, Phillips M, Tardif JC. Informed consent in the context of pharmacogenomic research: ethical considerations. Pharmacogenomics J. 2011;11:155–61. 21445091 10.1038/tpj.2011.11

[19] Haga SB, O’Daniel JM, Tindall GM, Lipkus IR, Agans R. Survey of US public attitudes toward pharmacogenetic testing. Pharmacogenomics J. 2012;12:197–204. 21321582 10.1038/tpj.2011.1 PMC3139751

[20] Diaz VA, Mainous AG III, Gavin JK, Wilson D. Racial Differences in Attitudes toward Personalized Medicine. Public Health Genomics. 2014;17:1–6. 24080914 10.1159/000354785

[21] Bevan JL, Lynch JA, Dubriwny TN, Harris TM, Achter PJ, Reeder AL, Condit CM. Informed lay preferences for delivery of racially varied pharmacogenomics. Genet Sci. 2003;5:393–9. 10.1097/01.gim.0000087989.12317.3f 14501835

[22] Patel MG, Dorward J, Yu L-M, Hobbs FR, Butler CC. Inclusion and diversity in the PRINCIPLE trial. Lancet. 2021;397:2251–2. 34119064 10.1016/S0140-6736(21)00945-4 PMC9752781

[23] Passero L, Srinivasan S, Roberts MC. Examining the role of language competency in genetic testing awareness among adults in the United States. J Genet Couns. 2022;31:1054–61. 35388538 10.1002/jgc4.1576

[24] Amato R, Del Toro-Pagan NM, Nguyen H, Plummer J, Pizzolato K, Krause D, Dowd D. Assessing the Impact of Simplified Language on a Patient-Facing Pharmacogenetic Report: A User Comprehension Study. J Pers Med. 2025. 10.3390/jpm15060247. 40559109 10.3390/jpm15060247 PMC12194072

[25] Bahramsoltani R, Rahimi R, Farzaei MH. Pharmacokinetic interactions of curcuminoids with conventional drugs: A review. J Ethnopharmacol. 2017;209:1–12. 28734960 10.1016/j.jep.2017.07.022

[26] Albassam AA, Ahad A, Alsultan A, Al-Jenoobi FI. Inhibition of cytochrome P450 enzymes by thymoquinone in human liver microsomes. Saudi Pharm J. 2018;26:673–7. 29989011 10.1016/j.jsps.2018.02.024 PMC6035319

[27] Li M, Chen P, Yue Q, Li J, Chu R, Zhang W, Wang H. Pungent ginger components modulates human cytochrome P450 enzymes in vitro. Acta Pharmacol Sin. 2013;34:1237–42. 23770984 10.1038/aps.2013.49 PMC4003154

[28] Whirl-Carrillo M, Huddart R, Gong L, Sangkuhl K, Thorn CF, Whaley R, Klein TE. An Evidence‐Based Framework for Evaluating Pharmacogenomics Knowledge for Personalized Medicine. Clin Pharmacol Ther. 2021;110:563–72. 34216021 10.1002/cpt.2350 PMC8457105

[29] PHARMGKB PHARMGKB:CYP2C9 Clinical Annotations. https://www.pharmgkb.org/gene/PA126/clinicalAnnotation. Accessed 6 Mar 2025.

[30] Nizamuddin S, Dubey S, Singh S, Sharma S, Machha P, Thangaraj K. CYP2C9 Variations and Their Pharmacogenetic Implications Among Diverse South Asian Populations. Pharmgenomics Pers Med Volume. 2021;14:135–47. 10.2147/PGPM.S272015 PMC7850565 33536773

