# Supplementary material for: Survey to inform personalised prescribing in a British South Asian community: pharmacogenomics and traditional medicine use
Source: BMC Med. 2026 May 15;24:387. doi: 10.1186/s12916-026-04914-9 (PMC13352602; doi:10.1186/s12916-026-04914-9)
Supplement: Supplementary file 1 — Supplementary Material 1: Additional File 1: Full Survey [file 12916_2026_4914_MOESM1_ESM.pdf]

Informed Consent

Consent Form

Thank you for your interest in this research. Should you wish to participate in the study, **please consider the following statements**. Please check the box next to **all or any of the statements that you agree with**.

By providing consent to the items below you are confirming that you have read and understood the participant information sheet and **are willing to participate in this research**, however you are reminded that **you are free to withdraw your participation** at any time.

If you have any questions please contact us by email at e.magavern@qmul.ac.uk. You can also refer back to the information sheet [here]

To participate in this study you must be at least 18 years old and of Bangladeshi or Pakistani ancestry.

Please check each box if you consent to the statement next to it

- ☐ I confirm that I have read the [Participant Information Sheet 1.0] for the above study; or it has been read to me.I have had the opportunity to consider the information, ask questions and have had these answered satisfactorily
- ☐ I understand that my participation is voluntary and that I am free to stop taking part in the study at any time without giving any reason and without my rights being affected.
- ☐ I understand that my data collected on the questionnaire will be accessed by the research team.
- ☐ I understand that my data will be securely stored stored on a confidential research database in accordance with the data protection guidelines of the Queen Mary University of London a 5-year period in pseudonymized form.
- ☐ I understand that I can access the information I have provided and request destruction of that information at any time prior to analysis. I understand that following data analysis I will not be able to request withdrawal of the information I have provided.
- ☐ I understand that the researcher will not identify me in any publications and other study outputs using personal information obtained from this study.
- ☐ I agree to take part in the above study.

Default Question Block

Thank you very much for participating. We would like your views on the following questions. The survey should not take more than 15 minutes to complete.

Different people respond differently to the same medicine. We're interested in your opinion about why that is. By medicine we mean medicine prescribed by a doctor or medicine that you buy yourself over the counter in the pharmacy.

Which of the below options can impact how you respond to a medicine?

Please select all that apply

- ☐ How old you are
- ☐ If you are a man or a woman
- ☐ How much you weigh
- ☐ Medical conditions you have
- ☐ Other medicines you take
- ☐ What you eat and drink
- ☐ The weather where you live
- ☐ Lifestyle choices (for example: smoking or drinking alcohol)
- ☐ Ethnicity
- ☐ DNA or Genetics
- ☐ How your family member responded to the same medication

- ☐ Taking the medication as prescribed
- ☐ None of the above

What do you think about this statement: DNA can influence your response to medicines

- ○ Strongly disagree
- ○ Disagree
- ○ Neither agree nor disagree
- ○ Agree
- ○ Strongly agree

Block 1

Experience with medicine

We're interested in your experiences around taking medicines

Have you ever taken a medicine but felt it did not work (prescription or over the counter medication)?

- ○ Yes
- ○ No

Have you ever had a side effect from a medicine (prescription or over the counter medication)?

- Yes
- No

Are you prescribed any medicines on a regular basis?

- Yes
- No

How many different prescription medicines do you take?

Block 2

Many people find a way of using their medicines which suits them. This may differ from the instructions on the label or from what their doctor had said. Here are some ways in which people have said they use their medicines.

For each statement, please click the option which best applies to you

I take less than instructed

- Always
- Often
- Sometimes
- Rarely
- Never

I stop taking it for a while

- Always
- Often
- Sometimes
- Rarely
- Never

I miss out a dose

- Always
- Often

- Sometimes
- Rarely
- Never

I alter the dose

- Always
- Often
- Sometimes
- Rarely
- Never

I forget to take it

- Always
- Often
- Sometimes
- Rarely
- Never

Block 3

Many people find a way of using their medicines which suits them best. They may also choose to add traditional or herbal remedies (homemade or over the counter)

alongside, or instead of, medicines prescribed by their doctor.

Do you use any of the following traditional or herbal remedies?

Please select all that apply.

- ☐ Ashwagandha (Indian Ginseng/Indian Winter Cherry)
- ☐ Fennel (Saumf)
- ☐ Turmeric home remedies (Haldi)
- ☐ Ginger home remedies
- ☐ Shilajit
- ☐ Joshanda
- ☐ Psyllium husk (Isabgol/Isapgol)
- ☐ Senna
- ☐ Black seed (Kalonji/Kalojeera)
- ☐ Other
- ☐ I do not use any traditional or herbal remedies

How often do you use these traditional or herbal remedies?

- ○ Never
- ○ Occasionally

- Sometimes
- Often
- Always

How do you use these traditional or herbal remedies?

- Alongside my prescribed medication(s)
- Instead of my prescribed medication(s)
- I do not use them

Block 4

DNA testing to personalise medicine choice

We would like to know what you think about being offered DNA testing to see which medicines and dose would suit your body. The information in your DNA might tell doctors that you are more or less likely to benefit from a medication or to have a side effect from a medication. DNA is passed down from your parents to you before you are born and does not change throughout your life. The test would not hurt and could be done from a routine blood test or “spit” sample.

This test will not tell you if you have a medical problem now or if you will have a medical problem

in the future, only how you may react to a medication.

This image illustrates the use of DNA testing to personalise medication choice.

Infographic reproduced with permission from Royal College of Physicians and British Pharmacological Society. Personalised prescribing: using pharmacogenomics to improve patient outcomes. Report of a working party. London:RCP and BPS, 2022

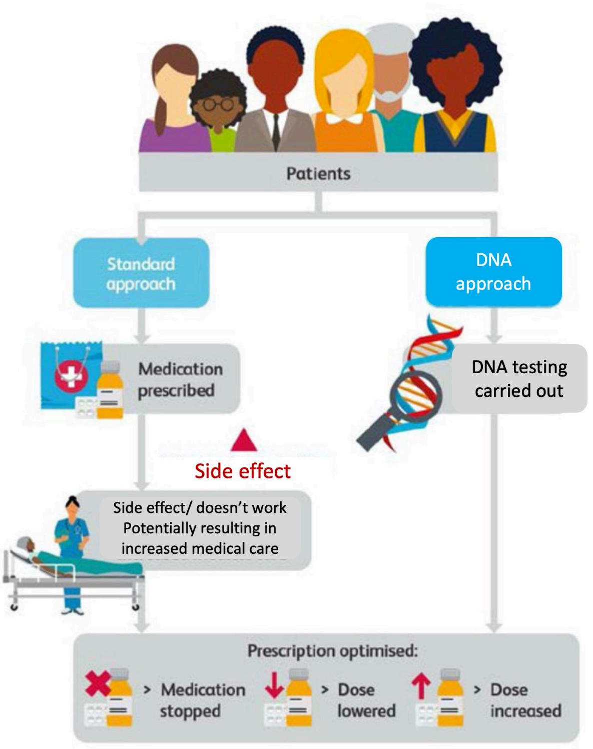

I would like to give a DNA sample to help decide if a medicine will suit me (for example a different dose or medicine might be better for me).

○ Strongly disagree

- Disagree
- Neither agree nor disagree
- Agree
- Strongly agree

I would be more likely to take medication as instructed by my doctor if DNA results suggested the medicine would suit me

- Strongly disagree
- Disagree
- Neither agree nor disagree
- Agree
- Strongly agree

This test will not tell you if you have a medical problem now or if you will have a medical problem in the future, only how you may respond to a medicine

I would have concerns about taking this DNA test

- Strongly disagree
- Disagree

- Neither agree nor disagree
- Agree
- Strongly agree

Please explain why you agree or disagree

I would encourage my family and friends to have this DNA test to guide medicine use

- Strongly disagree
- Disagree
- Neither agree nor disagree
- Agree
- Strongly agree

Block 5

Where DNA information is stored

We would like to understand where DNA information (to see which medicines and doses would suit your body best) should be stored.

I would like to keep this information about how I might respond to medications with me, for example on my phone, and be able to give it to any health care providers who treat me

- Strongly disagree
- Disagree
- Neither agree nor disagree
- Agree
- Strongly agree

I would like this information from my DNA about how I might respond to medication to be kept in hospital and GP NHS healthcare records

- Strongly disagree
- Disagree
- Neither agree nor disagree
- Agree
- Strongly agree

Block 6

Data sharing for research purposes

We would like to know how you would feel about sharing DNA and clinical information about medicines for research. This research would help to make medicines safer and more effective.

I would be willing to share information from my DNA about how I respond to a medicine with:

Please select all that apply

- ☐ Academic researchers (for example researchers from universities)
- ☐ The UK medicine regulators (The Medicines and Healthcare products Regulatory Agency (MHRA)). This agency is responsible for making sure that medicines available in the UK are safe and work well.
- ☐ Pharmaceutical industry researchers (working for privately owned organisations that develop and make medications)
- ☐ I would not want to share this information for research

I would be more comfortable sharing this DNA and clinical information for research if I knew a faith leader had done so

- Strongly disagree
- Disagree
- Neither agree nor disagree
- Agree
- Strongly agree

I would be more comfortable sharing this DNA and clinical information for research if I knew my family or friends had done so

- Strongly disagree
- Disagree
- Neither agree nor disagree
- Agree
- Strongly agree

Block 7

Views about data misuse

We want to know your views about DNA data misuse. This is to identify any concerns and think about solutions.

Please note that we are talking about testing DNA that will

only tell you how you might respond to a medication. It will NOT tell you if you have a disease or a risk for disease.

I'm concerned about potential misuse of my DNA information

- ○ Yes
- ○ No

I'm concerned about potential misuse of my DNA information by

Please select all that apply

- ☐ Healthcare practitioners
- ☐ Academic researchers (for example researchers from universities)
- ☐ Pharmaceutical industry/companies (privately owned organisations that develop and make medications)
- ☐ Other

I would want stronger protections on this DNA information about medicine response than other health information (for example medical problems, scans, or which medications you've been prescribed)

- Yes
- No

Please explain why you would want stronger protections and what protections you would like

Block 8

Communication, Outreach and Education

Educating patients about this use of DNA testing (to tailor medication choices rather than to look for disease) is important.

- Strongly disagree
- Disagree
- Neither agree nor disagree
- Agree
- Strongly agree

How would you like this information about DNA testing for medicine response to be available?

Please select all that apply

- ☐ Leaflets from GP surgery/hospital clinic
- ☐ Posters at GP surgery/hospital clinic
- ☐ Podcasts
- ☐ Videos
- ☐ On social media such as TikTok, Instagram, Facebook, X (formerly twitter)
- ☐ Whatsapp groups
- ☐ Mosque
- ☐ Community Centres
- ☐ Schools
- ☐ Shopping malls
- ☐ Other
- ☐ I do not want this information

Block 9

Now we would like to know about any concerns that British-Bangladeshi and British-Pakistani communities may have

There are concerns unique to the Pakistani or Bangladeshi ancestry population within the UK about this use of DNA to personalise medicines

- Strongly disagree
- Disagree
- Neither agree nor disagree
- Agree
- Strongly agree

Please explain your answer to the above question

Block 10

National reporting system for side effects

Have you heard of the Yellow Card reporting system that allows you to report medication side effects?

- Yes
- No

Did you know that anyone can submit a Yellow Card report to the UK medicines regulators to report a bad reaction to a medication?

- Yes
- No

Block 11

Now we would like you to tell us a little bit more about yourself to understand different views

How old are you?

Gender

- ○ Male
- ○ Female

I've completed education at the level of

- ○ No schooling completed
- ○ Primary school
- ○ Secondary school
- ○ Technical college qualification
- ○ University degree

What country was your highest level of education completed in?

- ○ UK
- ○ Pakistan
- ○ Bangladesh
- ○ India
- ○ Other South Asian country
- ○ EU country
- ○ Other

The language I speak the most often is

- Balochi
- Bengali
- English
- Farsi
- Gujarati
- Hindi
- Kashmiri
- Punjabi
- Pashto
- Sindhi

I am in paid employment

- ○ Yes
- ○ No

Powered by Qualtrics
